# The Usage of Reversed Köle-Type Mandibular Anterior Segmental Subapical Osteotomy: Radiological, Clinical, and Surgical Considerations

**DOI:** 10.3390/diagnostics16162563

**Published:** 2026-08-14

**Authors:** Kamil Nelke, Krisztián Nagy, Angela Rosa Caso, Massimiliano Gilli, Ömer Uranbey, Maciej Janeczek, Agata Małyszek, Maciej Dobrzyński, Wojciech Pawlak, Klaudiusz Łuczak

**Affiliations:** 1 Maxillo-Facial Surgery Ward, EMC Hospital, Pilczycka 144, 54-144 Wrocław, Poland; 2 Academy of Applied Sciences, Health Department, Academy of Silesius in Wałbrzych, Zamkowa 4, 58-300 Wałbrzych, Poland; 3 Pediatric Center, Semmelweis University, 1083 Budapest, Hungary; nagykrisztian@me.com; 4 Department of Medicine, Section of Maxillo-Facial Surgery, University of Siena, Viale Bracci, 53100 Siena, Italy; 5 Department of Maxillo-Facial Surgery, Hospital of Perugia, Sant’Andrea Delle Fratte, 06132 Perugia, Italy; 6 Department of Oral and Maxillofacial Surgery, Faculty of Dentistry, Aydın Adnan Menderes University, 09010 Aydın, Türkiye; 7 Department of Biostructure and Animal Physiology, Wrocław University of Environmental and Life Sciences, Kożuchowska 1, 51-631 Wrocław, Poland; maciej.janeczek@upwr.edu.pl (M.J.);; 8 Department of Pediatric Dentistry and Preclinical Dentistry, Medical University in Wrocław, Krakowska 26, 50-425 Wrocław, Poland

**Keywords:** subapical osteotomy, MASSO, reversed Köle-type modification, orthognathic surgery, anterior segmental osteotomy, Köle osteotomy, cone-beam computed tomography

## Abstract

The currently common procedures in orthognathic surgery are widely used. Different forms of skeletal malocclusion can be treated with great outcomes in terms of bite, bone harmony, function, improved mastication, esthetics, and even decreased problems with breathing. Each type and intensity of malocclusion may affect decision-making in terms of distraction, selection of osteotomy design, or even guide clinical and surgical decisions to use a less popular osteotomy protocol. CBCT (cone-beam computed tomography) is currently a milestone in the establishment of the condition of jaw bones, bone structure, temporomandibular joints, and central relation (CR), as well as the scope of maxilla–mandibular and tooth relations. When establishing all of the following in a clinical and radiological (CBCT-based) evaluation, it becomes possible to measure the occlusal plane, height, length, and three-dimensional position of each dental segment, as well as other dentoalveolar features. In the present case, CBCT helped distinguish a localized vertical anterior mandibular dentoalveolar discrepancy from generalized mandibular malposition and supported the planning of a mandibular anterior segmental subapical osteotomy (MASSO) by mapping the osteotomy corridor in relation to the dental roots, cortical bone, periapical bone height, and neurovascular structures. Significant therapeutic complexity emerges when severe skeletal Class III malocclusion (mandibular prognathism) is compounded by transverse maxillary discrepancies and a markedly elevated mandibular anterior segment. This constellation of deformities leads to profound disturbances in the anterior facial height, a disrupted maxillomandibular interincisal relationship, and the loss of a level occlusal plane. The presented case highlights how the patient’s clinical evaluation should be carefully compared with radiological data, status of both cortices in the mandible, the height of the mandibular anterior segment, presence of gum recessions and poor bone status that may affect healing, osteotomies, and occurrence of some unwanted events. Therefore, CBCT was useful not only for diagnosis and planning, but also for understanding the biological limits of movement and the risk of postoperative periodontal complications in this uncommon reversed Köle-type MASSO.

**Figure 1:**
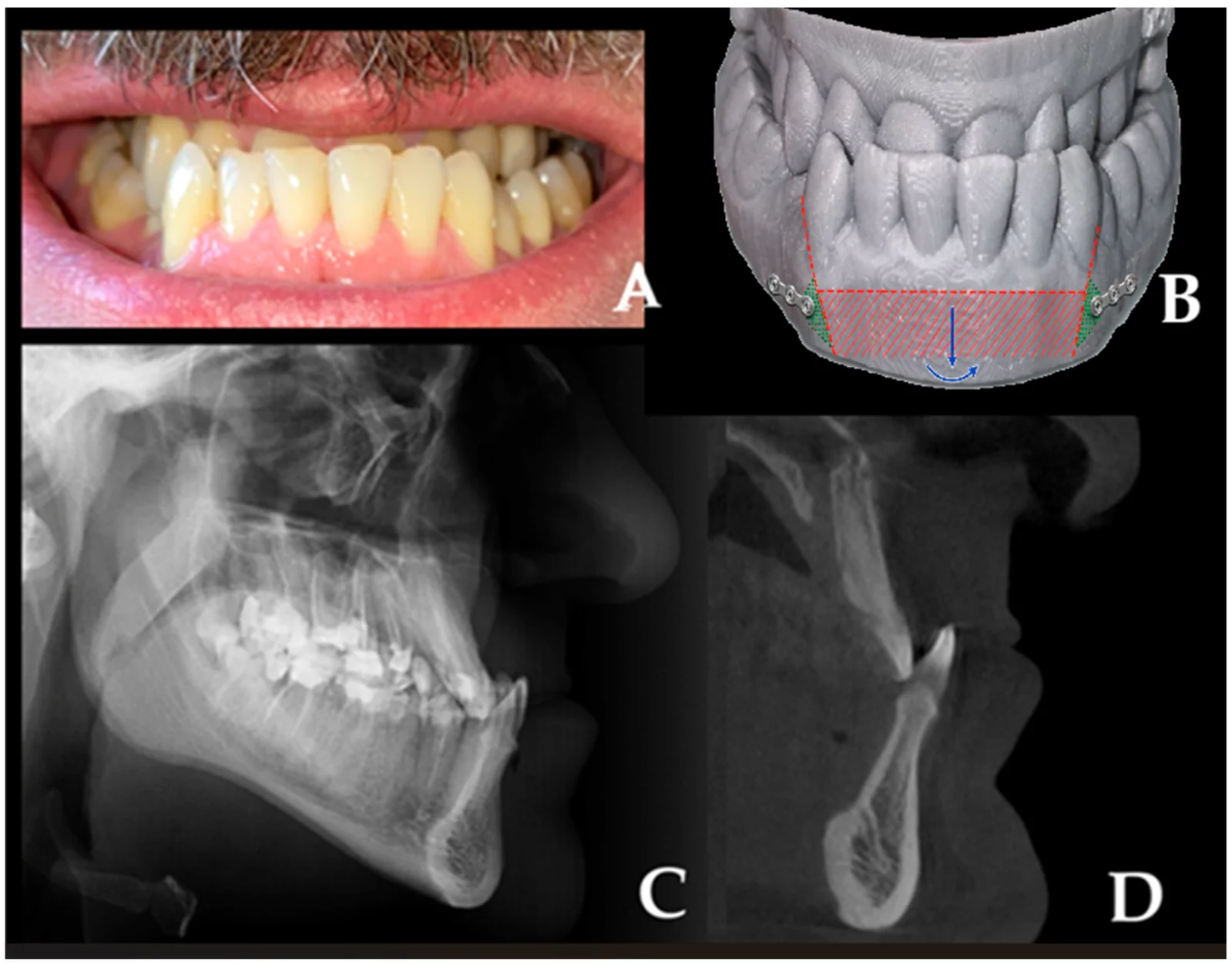
There are many types of dental malocclusion that can be easily treated with some basic orthodontic appliances. The scope of each skeletal malocclusion, on the other hand, quite commonly requires a collaboration between maxillofacial surgeons and orthodontists. Etiology, severity, and oral, dental, and facial characteristics in each case might have some typical or atypical features. The disproportions in bone shape, volume, position, and three-dimensional relations affect the scope of possible approaches and levels of the scheduled treatment plan. Most commonly, typical osteotomies used in orthognathic surgery (OS) are related to the scope of maxillary bone osteotomy according to the Le Fort I principle, as well as bilateral sagittal split osteotomy (BSSO) of the mandible [1,2,3]. Each osteotomy has its own modifications, alterations, and other specially designed scopes of surgical cuts, osteotomies, ostectomies, or others. Because of recent advances in three-dimensional cone-beam computed tomography (CBCT) as well as individual patient solutions (IPSs) and virtual planning, great improvements in the scope of planning and processing with surgeries are known. In complex segmental deformities, CBCT-based analysis may help distinguish whether the discrepancy is generalized skeletal malposition or a localized dentoalveolar problem requiring a more selective osteotomy design. Each OS not only affects bite, skeletal relation, and facial esthetics, but also function and stability of TMJ (temporomandibular joints), as well as other aspects, like the positive effect on airways, decreasing snoring, and OSAS (obstructive sleep apnea syndrome) symptoms [3]. Nowadays, some atypical and “old-school-based” osteotomies are not that common; however, they can greatly affect patients’ outcomes and final results if they are planned based on a novel virtual, CBCT-based IPS protocol. Mandibular segmental osteotomies may involve anterior, lateral, or total dentoalveolar segments depending on the location and extent of the deformity [1,2,3,4,5]. The anterior mandibular procedure has historically also been termed “subapical mandibular osteotomy,” “anterior subapical osteotomy,” or the “Köle procedure”; for consistency, the term “mandibular anterior segmental subapical osteotomy (MASSO)” is used throughout this report. The following radiological images represent the situation when the upper incisor crown is almost entirely covered by the lower incisors, while both lateral segments remain on the same level in both clinical and 3D-printed model evaluation (**A**,**B**); the red line marks the scope of anterior segment elevation in the presented skeletal malocclusion that requires osteotomy and its down-positioning, marked as red–green markings. This finding is important because it suggests that the main vertical discrepancy is concentrated in the anterior mandibular dentoalveolar segment, whereas the lateral mandibular segments preserve a more acceptable occlusal level. On the other hand, very valuable information is addressed by a routine cephalometric radiograph and CBCT in the sagittal plane in order to assess the scope of occlusal plane tilting, dentoalveolar relations, and the scope of anterior mandibular tooth-segment elevation towards the lateral mandibular aspects (**C**,**D**). Therefore, CBCT was useful not only for diagnosis and planning but also for understanding the biological limits of movement and the risk of postoperative periodontal complications associated with this uncommon reversed Köle-type MASSO.

**Figure 2:**
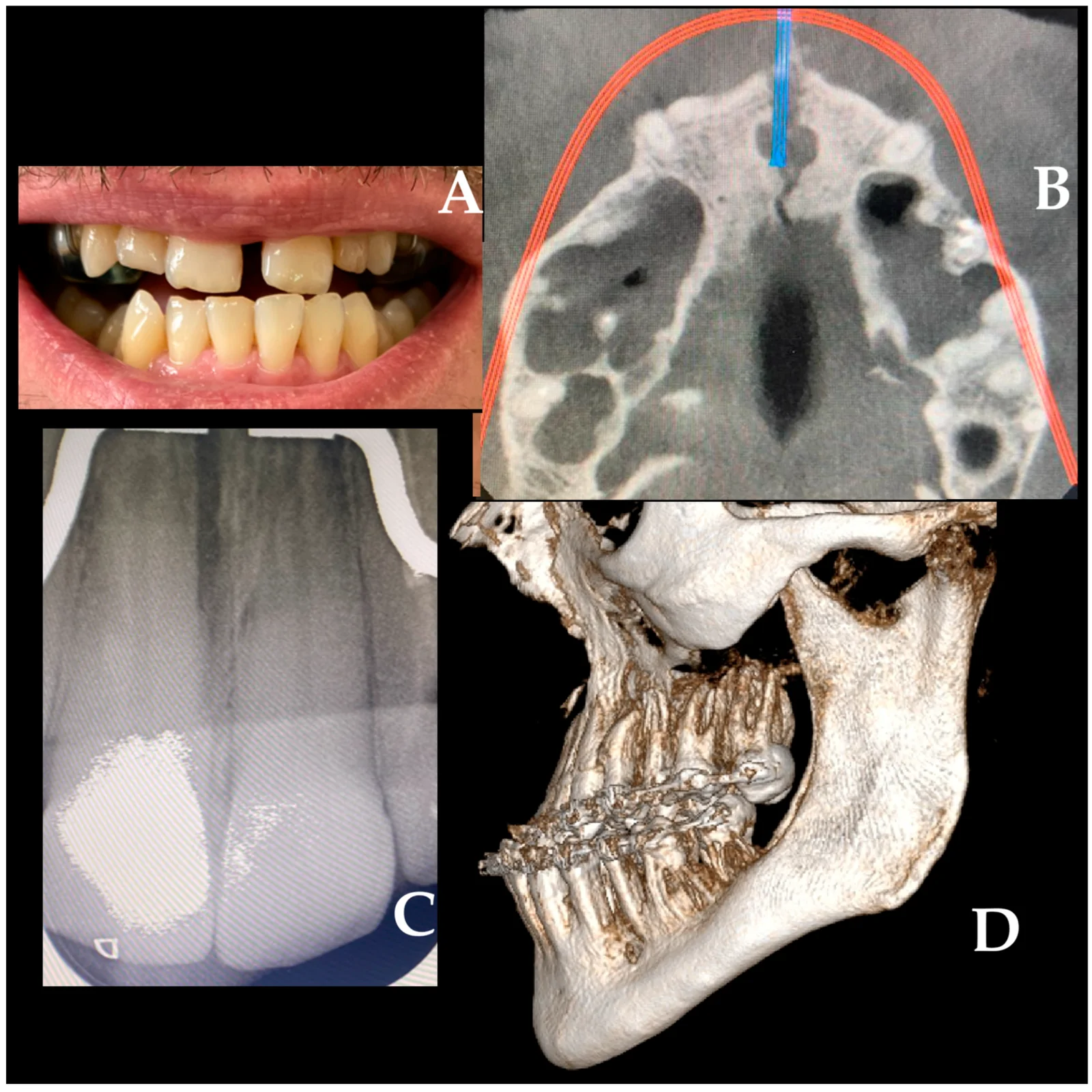
During the preparation for the correction of skeletal malocclusion, the patient underwent necessary dental treatment. A typical surgically assisted rapid palatal expansion (SARPE) using a Hyrax appliance and general anesthesia was necessary to expand the maxillary bones and correct maxillary narrowing and loss of its transverse dimension (**A**–**C**). Further orthodontic preparation was uneventful. At the time of patient readiness for the main OS, a problem arose because of an increased occlusal height in the anterior mandibular segment. Typically, this might have been overcome with segmental maxillary osteotomy; however, the patient’s lack of transverse dimension was a problem. Secondly, there was no possibility to apply any orthodontic forces to intrude the mandibular anterior teeth (**D**). Another main reason that was an unfavorable factor was the poor cortical bone condition of the anterior mandibular teeth, gingival recession at the canines, and the patient’s age of over 45 years. CBCT evaluation was important at this stage because it showed that the discrepancy was mainly concentrated in the anterior mandibular dentoalveolar segment, while the right and left lateral mandibular segments were in more acceptable alignment with the maxillary occlusal plane. Because the right and left lateral mandibular segments were in proper alignment with the maxillary bones and had similar height of the occlusal plane, a decision was made to schedule a reversed Köle-type MASSO involving teeth 34–43 (FDI notation) to lower the bone fragment and decrease its superior protrusion and unwanted rotation. The reversed Köle-type MASSO was performed, with bone grafts placed along the lateral vertical osteotomy gaps to support healing and stabilization of the segment in its new position. One of the uses of MASSO for the correction of mandibular incisor axis, rotation, extrusion, and occlusal plane was presented by Sahin et al. [6]. The key radiological issues that should be addressed before planning for MASSO are: evaluation of the alveolar bone surrounding each tooth between the planned vertical bone cuts, measurement of interradicular spaces, assessment of root angulation, root apices, and periapical bone height, assessment of the minimum distance between the planned vertical and horizontal osteotomy lines and the dental roots, evaluation of the thickness and condition of both buccal and lingual cortices, evaluation of the angulation of canines and premolars in terms of safe osteotomy (approximately 3 mm of safety distance is desirable, when anatomically possible), establishment of the position and elevation of the mental foramen, measurement of the height of the anterior mandibular tooth periapical area towards the anterior mandibular basis in case simultaneous genioplasty (chin advancement or reduction) is planned, and also assessment of the presence of accessory mental foramina, anterior loop, labial foramina, lingual canals, gonial canals, or mylohyoid/sublingual vascular canals in the operated field to avoid unwanted bleeding and possible hematoma formation in the floor of the mouth [6,7,8,9,10,11,12,13,14,15]. In addition, CBCT evaluation of thin cortices, fenestration/dehiscence tendency, existing gingival recession, and reduced periodontal reserve may help explain why postoperative soft-tissue or periodontal complications can occur despite adequate surgical execution.

**Figure 3:**
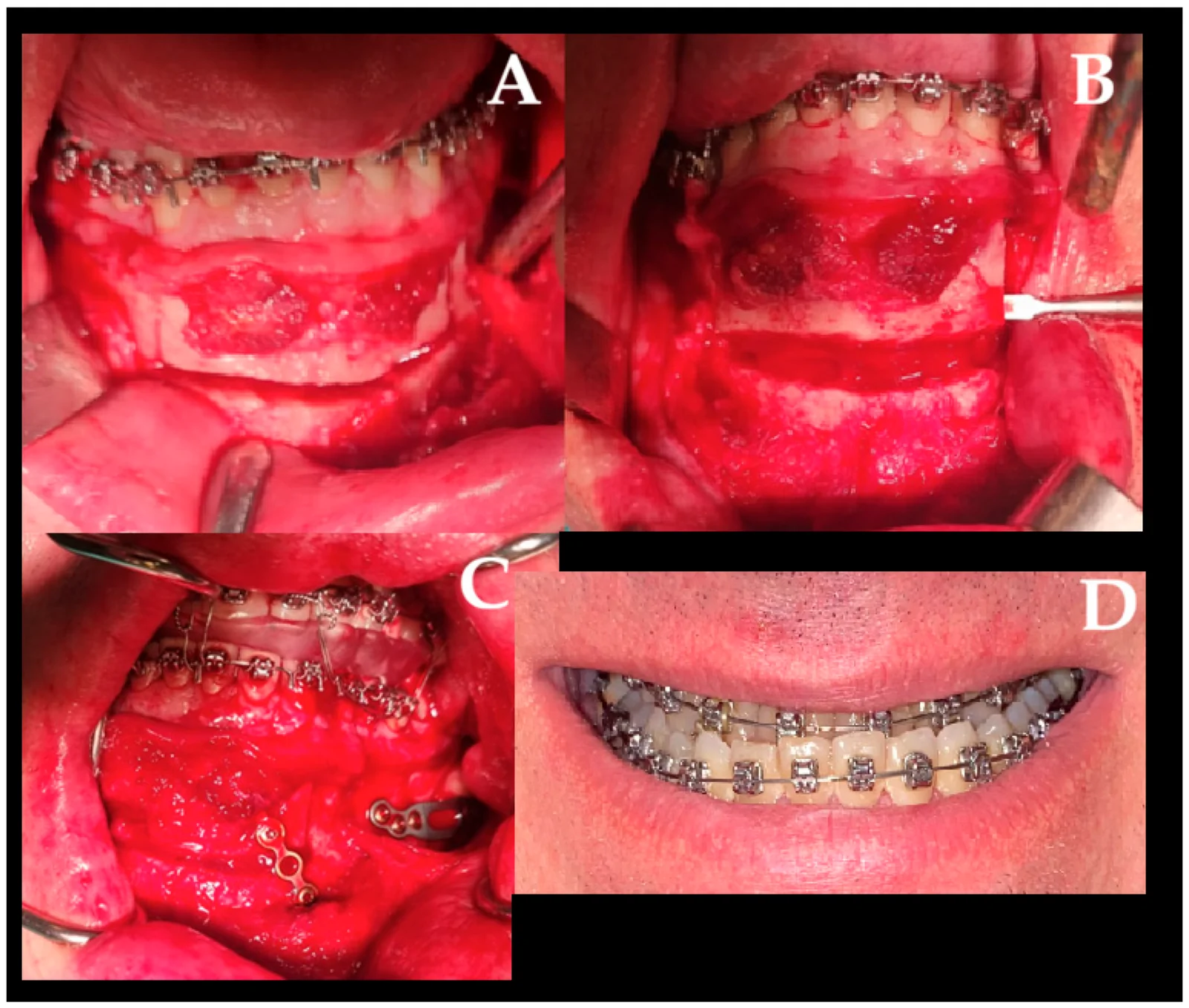
In the field of OS, a lot of different types of possible osteotomies can be done. One of the quite interesting and yet not completely forgotten mandibular osteotomies is the subapical mandibular osteotomy (SMO), first mentioned by Hullihen in 1848, and later more popularized by Heinrich Köle in Graz (Austria). It is currently often referred to by a simple acronym known as ASO (anterior subapical osteotomy) [7,8]. Firstly presented in 1959, the mandibular anterior segment (MAS) surgery was aimed at treating mild-to-moderate skeletal open-bite deformities, as well as some forms of asymmetrical segmental deformities, especially in the mandible, and could be combined with premolar removal or could be accomplished with vertical interdental osteotomies [9,10]. Primary ASO was just reserved for the anterior mandibular segment, but currently, other modifications can also be used with it without additional genioplasty, BSSO, or other osteotomies, and with or without the necessity for bone grafts and additional plating devices [6,7,8,9,10]. Some authors combine anterior or total subapical osteotomies for the correction of class II division I incisor malposition, where the chin is well situated or needs additional grafting, when the pogonion position needs improvement, when anterior vertical facial height needs improvement or reduction, in some scope of mandibular vertical alveolar discrepancies, where OS is not used and MASSO is a valuable option, in some lateral open bites or partial anterior open bite, or when the disturbances in soft tissues in mentolabial angle/sulcus are found [6,7,8,9,10,11,12,13,14,15]. Although anterior mandibular subapical osteotomy can be adapted to different three-dimensional movements, the term “conventional Köle procedure” in this manuscript refers specifically to its classic application for correction of an anterior open bite. In that application, the mobilized anterior dentoalveolar segment is elevated or superiorly repositioned to bring the mandibular incisors into occlusion, and the resulting inferior gap may be filled with a bone graft [7,8,11]. In the present patient, the anterior mandibular dentoalveolar segment was positioned excessively superior to the lateral mandibular occlusal plane. The reversed Köle-type MASSO involving teeth 34–43 was approached through a limited vestibular subperiosteal tunnel, with soft-tissue elevation confined to the buccal osteotomy corridors to minimize disruption of the periosteal attachments and segmental vascular supply. The vertical interdental osteotomies were positioned at the lateral boundaries of the planned segment according to the CBCT-defined root corridors, while the horizontal subapical osteotomy was placed below the root apices, preserving the surrounding alveolar and basal bone. The osteotomy lines were initiated using a piezosurgical device and completed carefully with bone chisels under continuous irrigation. Rotary instruments were then used to remove the bony interference immediately inferior to the horizontal osteotomy and create sufficient space for downward repositioning. Following complete mobilization, the segment was translated inferiorly and rotated in the sagittal plane until the anterior and lateral mandibular occlusal levels were harmonized. No fixed osseous hinge was preserved; the final segment position was determined by passive seating of the surgical splint and achievement of stable occlusion. The residual lateral osteotomy gaps were grafted, and the segment was stabilized using titanium plate-and-screw fixation. Orthodontic ligatures and intermaxillary elastics provided additional support during early healing. Panels (**A**,**B**) demonstrate preparation and mobilization of the osteotomized segment, while panel (**C**) shows its final position and rigid fixation. Thus, “reversed” describes the inverse vertical movement and bone-removal pattern relative to the classic open-bite application and does not imply that the underlying osteotomy design itself is entirely novel. Alternative directions of anterior segmental movement, including incisor-axis correction and anteropositioning/proclination, have previously been described [6,15]. Therefore, the term “reversed” is used here to define a case-specific directional modification rather than to claim a previously unreported osteotomy design. While assessing the CBCT the following factors have to be studied: (1) tooth angulation and its root placement; (2) distance from the mandibular lower border to the tooth apices; (3) calculating at least 1 mm from the root and at least 2–3 from the periapical region; (4) measuring the mandible bone height, transverse and vertical dimensions to prepare sufficient burrs and chisels; (5) thickness of the cortices (buccal, lingual); (6) evaluating the distances between canine and premolar teeth to properly position the osteotomy lines; (7) evaluating the distance between two adjacent roots and then either using burrs/piezo surgery or bone chisels for a safer bone split. The following can be done with a surgical caliper and measurements or by using 3D-based individual cutting guides, especially recommended in very narrow bone corridors and tooth crowding. This step shows the transfer of the radiological and model-based plan into the surgical field, where bone removal, segment rotation, and fixation had to be coordinated to avoid unwanted occlusal canting or relapse. Given that MASSO is relatively uncommon and infrequently utilized in routine clinical practice, it is crucial to recognize that a misalignment of the mandibular lateral and anterior occlusal planes can result in severe occlusal and dental relationship disturbances (**D**).

**Figure 4:**
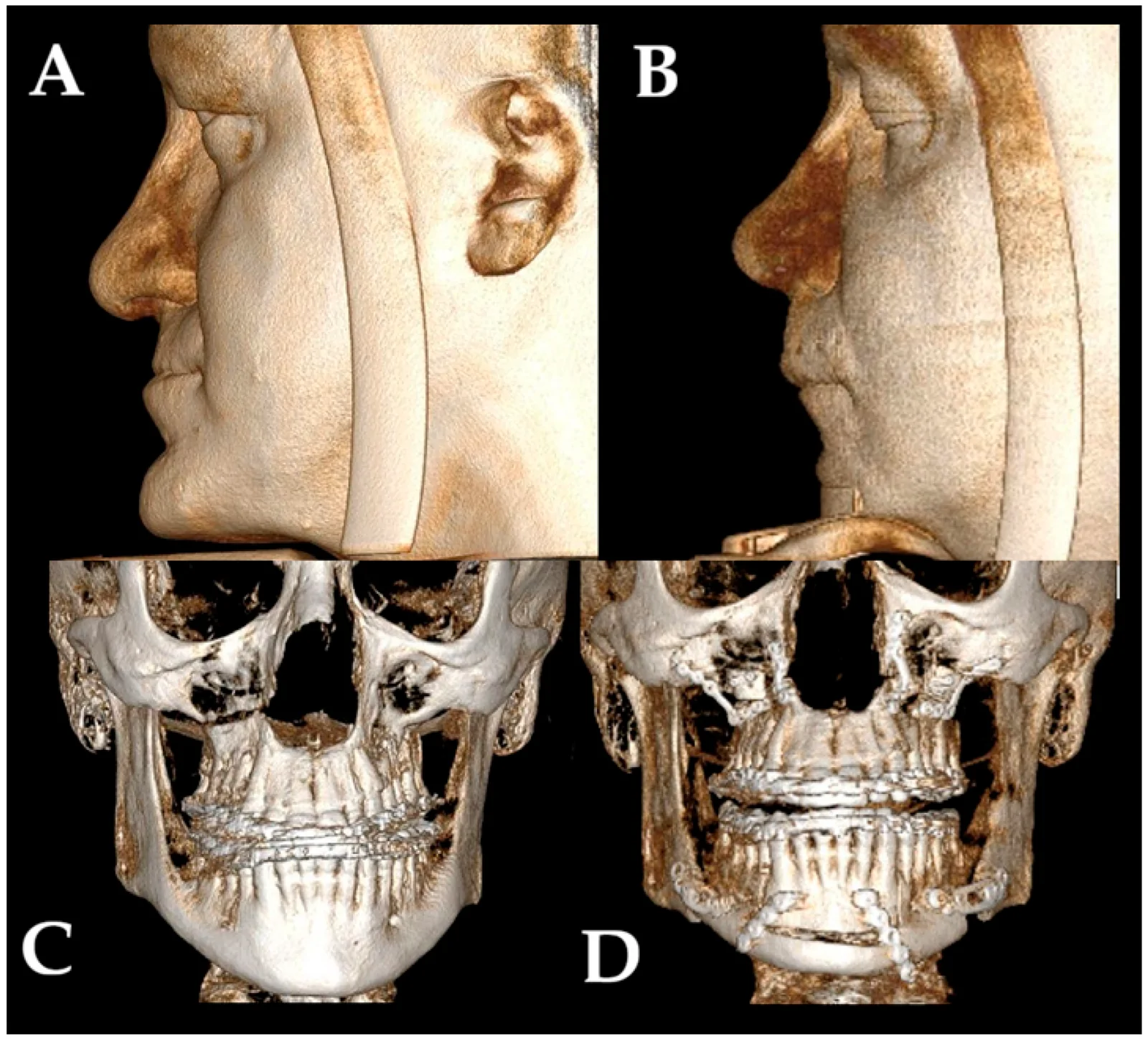
The typical MASSO by Köle (1959) [7] was used to close an anterior open bite. In the authors’ reversed modification, it improved the incisors’ dentoalveolar relation and leveled the occlusal level. Typical MASSO improved the mentolabial sulcus and required some bone grafting material to increase the vertical height [8,9,10,11,12]. Postoperative clinical and radiological images (**A**–**D**) demonstrate approximation of the anterior and lateral mandibular occlusal levels and the associated change in the anterior facial and mentolabial contours. From the perspective of OS and the esthetics and male characteristics of the lower face, in this case, a reduction in the mentolabial sulcus, correction of the protruded chin, and improvement of the mandibular occlusal level were necessary [6,7,8,9,10,11,12,13,14]. In other cases of retruded chin and non-prominent pogonion, a typical genioplasty with bone grafting material could be used in combination with MASSO if needed. In the present case, the postoperative clinical images demonstrated closure of the lateral open bite; however, visible gingival recession was noted in the region with pre-existing cortical deficiency. Because standardized tooth-specific pulp-sensibility testing and periodontal measurements were not available, no quantitative claim regarding pulp vitality or the magnitude of recession is made. This point is clinically important because CBCT-based evaluation of the periodontal envelope may show thin buccal or lingual cortices, fenestration/dehiscence tendency, and reduced periodontal reserve before surgery [9,10,11,12,13]. Therefore, postoperative recession should not always be interpreted as a purely technical complication, but also as a possible consequence of limited pre-existing bone and soft-tissue support. Despite the limited soft-tissue elevation used to preserve segment vascularity, the pre-existing periodontal limitations could not completely prevent postoperative recession. Perhaps when simultaneous free connective tissue grafting and onlay bone grafting were used, this might not happen or could be greatly reduced when occurring. Many approaches, techniques, and scope of surgical approaches can be applied in similar cases of increased anterior vertical facial height or in cases of its short or long face syndromes, as well as to improve proper bone volume and shape, according to male or female lower facial height characteristics [15,16,17,18,19,20]. The postoperative images demonstrate the altered occlusal relationship and soft-tissue contour following the combined MASSO, BSSO, and Le Fort I osteotomies, while the radiographs show maintained segment continuity and fixation at the available follow-up. Crucial pulp vitality is possible to maintain when longitudinal cuts from tooth roots with a minimum of 1 mm of surrounding bone are sustained [8,9,10,11,12]. However, when anatomy allows, a wider safety distance from dental roots remains preferable. It is worth pointing out that in reduction in bone height, no bone grafting is necessary; however, when it would require any extrusion, then bone grafts are necessary. When anterior mandibular segment axis correction might require adjunctive bone ostectomy and burr modeling with or without bone grafting in bone gaps, any possible scenario is possible. When planning an anterior, lateral, or total subapical osteotomy, the shape, position, and angulation of the mental foramen and inferior alveolar nerve affect the scope of osteotomy line presence. Nowadays, individual patient-specific cutting guides can be used with great value for minimizing nerve damage. In cases where formal virtual planning or patient-specific guides are not used, CBCT and 3D model evaluation may still help anticipate the final segment position, fixation requirements, and soft-tissue limitations [16,17,18,19,20,21,22]. The presented pre- and postoperative CBCT scans were made with a 3D-CBCT 20 × 20 FOV (field of view) imaging protocol based on a RayScan S 5471.3 mGy (RayCompany Ltd., Samsung 1-ro, Hwaseong-si, Gyeonggi-do, Republic of Korea).

**Figure 5:**
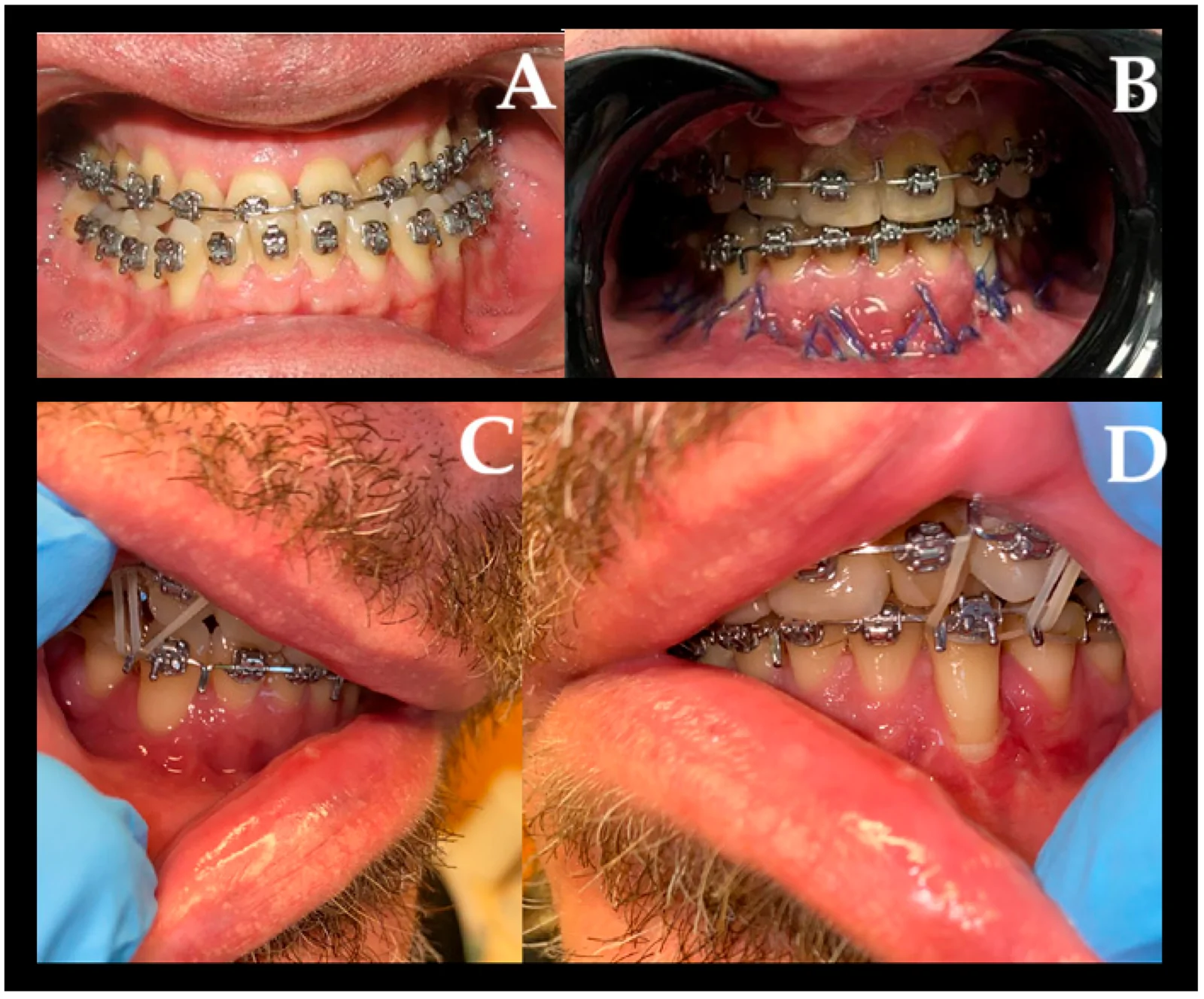
Initial occlusion demonstrated distinct anterior and lateral mandibular occlusal levels (**A**). The postoperative clinical image shows their visual alignment following MASSO (**B**). In each scenario, typical elastic wires are used to maintain stable occlusion and support the repositioned mandibular anterior segment during early healing. This postoperative view emphasizes that the aim of the reversed Köle-type MASSO was not only segment movement, but also stable occlusal-plane harmonization between the anterior and lateral mandibular segments. Other aspects might include some cleft cases or when an ortho-surgical compromise treatment is considered with a minimally invasive approach; correction of the elevated or sunken MASSO bone fragments; for MASSO advancement with or without a combination of BSSO/Le Fort I osteotomy with or without bone grafting; and should be considered in bimaxillary protrusion with or without a corrective chin genioplasty as well, or even in some cases of edentulism and insufficient bone volume before prosthodontic treatment. Typically, a box mandibular segment is lifted to improve tooth occlusion, while in the inferior osteotomy gap a bone fragment is placed in order to promote better bone healing and maintain the mandibular segment in a stable position within the new desired bite and occlusion [11,12]. Its modalities might include removal of the inferior part of the horizontal osteotomy line in the subapical tooth area to lower the segment, rotate it, and re-stabilize it in the desired position in order to correct the occlusal plane and tooth position. The presented images underline how bone volume, its shape, and scope of skeletal malocclusion can be approached successfully with MASSO [13,14,15,16,17]. Some other bone protocols, including bone osteotomies, bone grafting, reshaping procedures, or other high-speed burr bone surgeries, can be used in a great modality of approaches in order to shape the chin and mandibular basis area in the desired position, width, length, and volume [21]. In this context, CBCT and model-based evaluation are useful not to repeat the diagnosis, but to confirm that the planned amount of inferior repositioning, rotation, and bony reshaping is surgically achievable and compatible with stable fixation. In each case, bone chisels, osteotomy burrs, piezo surgery, or other devices can be used with great success if sufficient water-cooling is provided and the osteotomy cuts are placed at a safe distance from tooth apices and roots. Many surgical maneuvers can be used in order to cut and later stabilize the bone in proper position and angulation [5,20,21,22,23,24]. (**C**,**D**)-comparable CBCT before and after surgery in coronal view.

**Figure 6:**
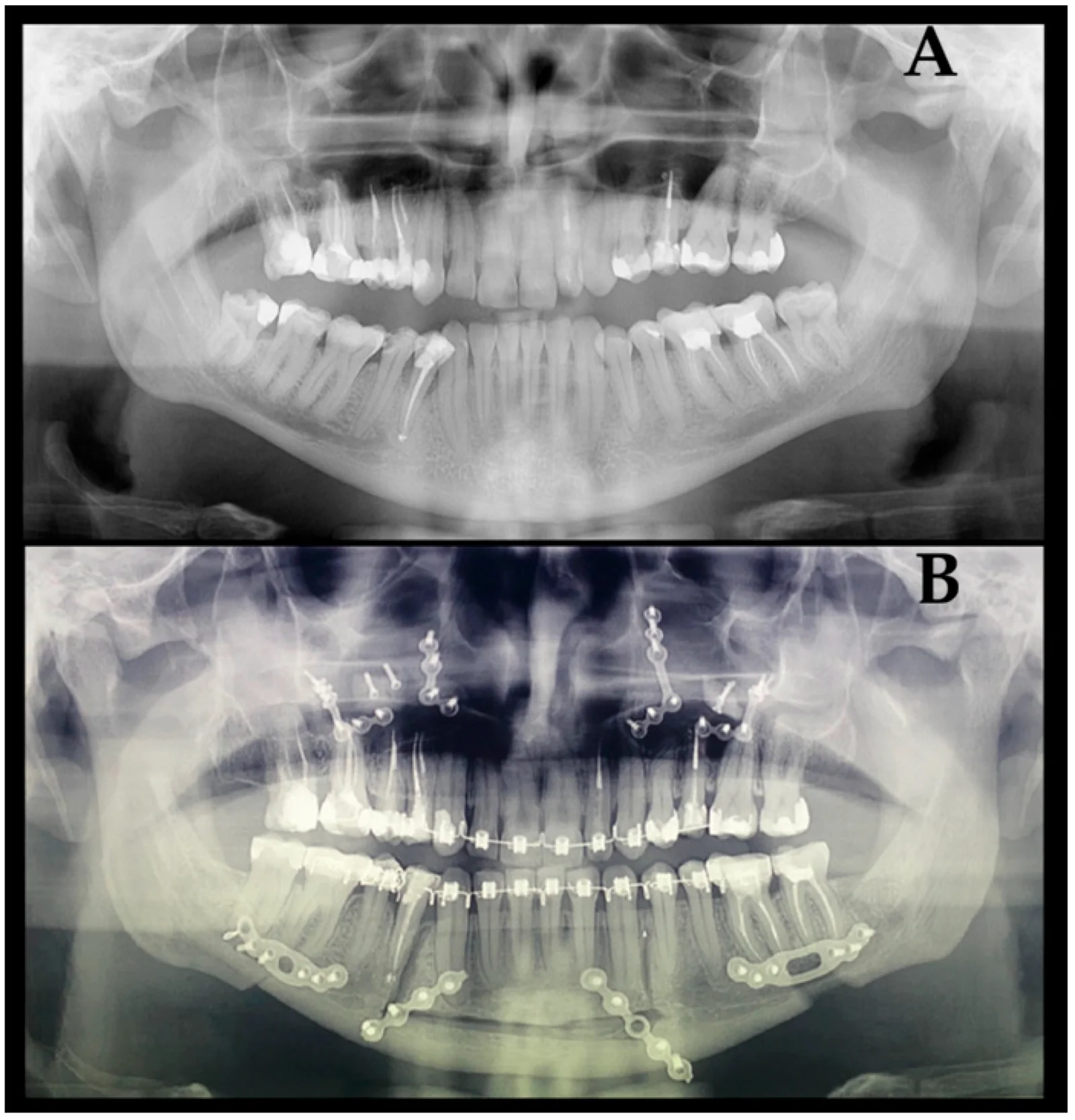
After each surgery, an OPG provides a good, quick, and quite sufficient radiological study to evaluate early outcomes from osteotomies and scope of bone cuts. On the other hand, if any disturbances in bone alignment, condyle head positioning or insufficient radiological appearance of tooth roots and their apices is found, then a CBCT should be done. Initial OPG revealed good radiological outcomes in terms of bone cut placements (**A**,**B**), proper bone fragments and osteosynthesis material placement, sufficient central condyle relation in the fossa, and absence of any unwanted bone fractures. Important clinical and radiological considerations for MASSO include changes in tooth angulation and vitality, segment displacement and rotation, bone grafting requirements, and the resulting effects on occlusion, anterior facial height, and the mentolabial region. Additionally, the oval, square, or trigonal-like mandibular and facial appearance can be achieved when the selected mandibular segments are moved, rotated, and positioned in the desired final position. On the other hand, the condition of cortices, status of alveolar bone, presence of good and healthy alveolar bone of at least 1 mm around the dental roots, lack of bone fenestration and bone loss, absence of pre-existing or treatment-related gingival recession (of either orthodontic, periodontal or combined origins), as well as the vitality of the periapical region (dental pulp vitality) and condition of the mental and/or inferior alveolar nerves should be evaluated before surgery. For this reason, CBCT should be considered as a preoperative risk-stratification tool rather than only a diagnostic image, especially when uncommon segmental movements are planned in the anterior mandible. Typical loss of sensation can have various stages, including paresthesia, hyper-/hypo-esthesia, and rarely other neuralgic pain episodes [8,9,10,11]. Surgical perspective should include the type of used burrs and saws to reduce bone overheating and support adequate water-cooling and appropriate plate positions with screws for granting adequate stability, minimizing tooth mobility, and promoting good bone healing [10,11,12,13,14,15,16]. Lastly, the bone segments should also require some steel ligature wiring with the rest of the dental braces/appliances to secure them additionally in the desired position, dental occlusion, and relationship to adjacent teeth, upper incisors, and scope of vertical and horizontal incisal overlapping. Thus, the final treatment plan should combine clinical occlusal evaluation, periodontal assessment, CBCT-based anatomical risk mapping, and intraoperative stabilization principles. Comparison of the preoperative and available early postoperative radiographs demonstrates maintained segment alignment and fixation on routine OPG imaging (**A**,**B**). However, while OPG is useful for global postoperative assessment, it cannot replace CBCT for preoperative evaluation of cortical thickness, root proximity, and neurovascular anatomy in complex mandibular subapical osteotomies.

**Figure 7:**
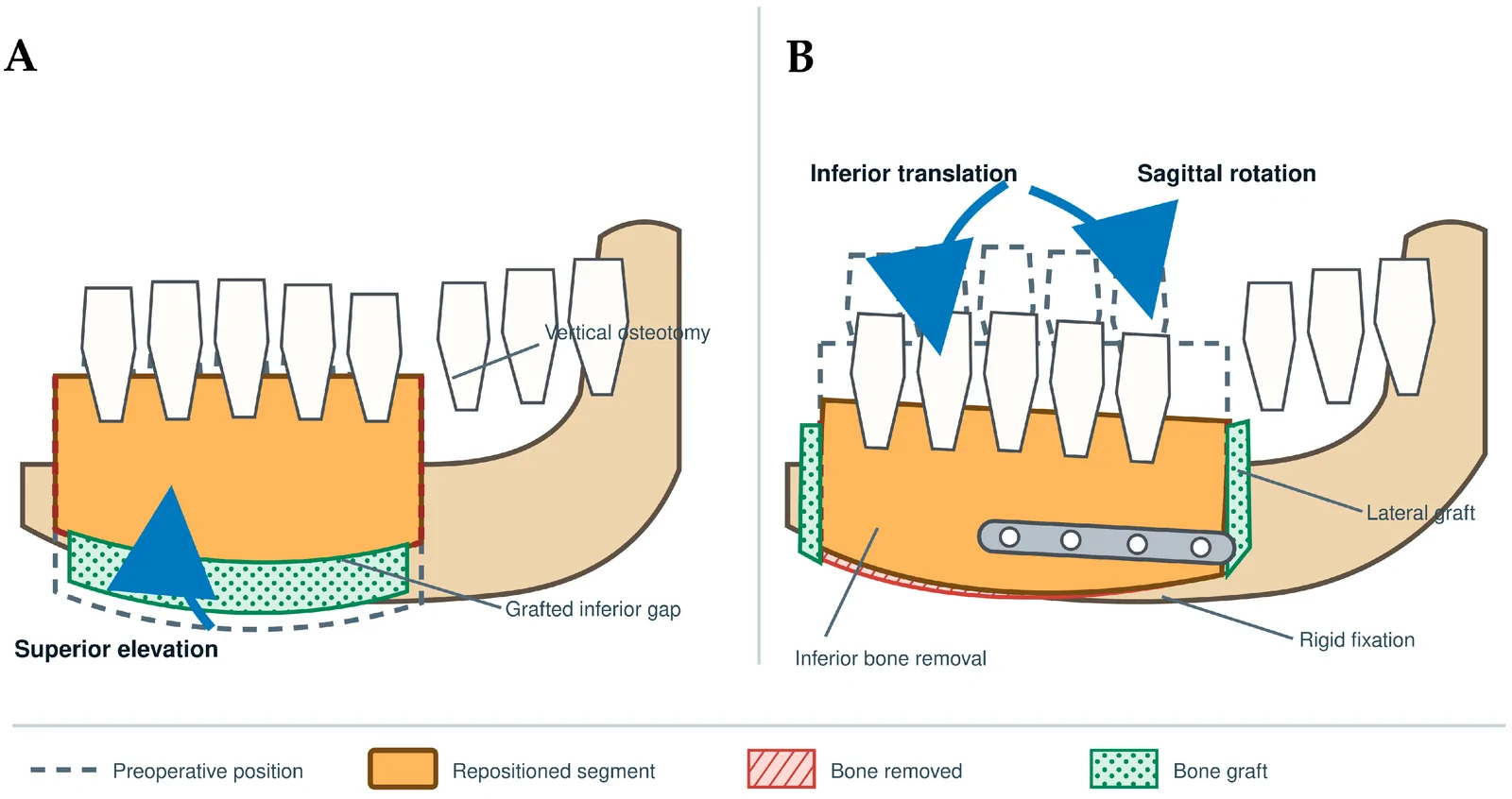
As a final conceptual synthesis of the radiological, periodontal, occlusal, and surgical considerations presented above, mandibular anterior subapical osteotomy should be regarded as a versatile, movement-oriented procedure rather than a technique restricted to a single historical indication. In addition to classical open-bite correction, it has been adapted for incisor-axis correction, dentoalveolar advancement, proclination, and combination with other orthognathic procedures [5,6,12,24]. In its conventional open-bite application, the mobilized anterior dentoalveolar segment is repositioned superiorly, with grafting of the resulting inferior gap when required [7,8,11,16]. This principle is illustrated in (**A**), where the upward blue arrow indicates superior movement and the green dotted area indicates the grafted inferior gap. In both panels, the gray dashed outline represents the preoperative segment position, the orange area its final position, and the red dashed boundaries the vertical and horizontal osteotomies. In the present patient, the excessively elevated anterior mandibular segment required the opposite vertical approach shown in (**B**). The red hatched area indicates the bony interference removed below the horizontal osteotomy, while the two blue arrows distinguish the dominant inferior translation from the accompanying sagittal rotation required to harmonize the anterior and lateral occlusal levels. These arrows indicate movement direction only and do not represent measured displacement or rotation around a fixed osseous hinge; the final position was determined by passive surgical-splint seating and stable occlusion. The green dotted lateral areas indicate bone grafting, and the gray plate and screws represent rigid fixation. Taken together, this final comparison demonstrates the case-specific use of reversed Köle-type MASSO for inferior repositioning and rotation of an elevated anterior mandibular segment.

## Data Availability

The data presented in this study are available on request from the corresponding authors due to patient privacy.
